# Characterization of Different *Capsicum* Varieties by Evaluation of Their Capsaicinoids Content by High Performance Liquid Chromatography, Determination of Pungency and Effect of High Temperature

**DOI:** 10.3390/molecules181113471

**Published:** 2013-10-31

**Authors:** Alberto González-Zamora, Erick Sierra-Campos, J. Guadalupe Luna-Ortega, Rebeca Pérez-Morales, Juan Carlos Rodríguez Ortiz, José L. García-Hernández

**Affiliations:** 1 Facultad de Agricultura y Zootecnia, Universidad Juárez del Estado de Durango, Km 35 Carretera Gómez Palacio-Tlahualilo, Ejido Venecia, CP 35111, Gómez Palacio, Durango, Mexico; E-Mails: agzfc@ujed.mx (A.G.-Z.); lupe_lunao@yahoo.com.mx (J.G.L.-O.); 2 Facultad de Ciencias Biológicas, Universidad Juárez del Estado de Durango, Av. Universidad SN, Fracc, Filadelfia, CP 35010, Gómez Palacio, Durango, Mexico; 3 Facultad de Ciencias Químicas, Universidad Juárez del Estado de Durango, Av. Artículo 123 SN, Fracc, Filadelfia, CP 35010, Gómez Palacio, Durango, Mexico; E-Mails: ericksier@gmail.com (E.S.-C.); rebecapms@ujed.mx (R.P.-M.); 4 Facultad de Agronomía, Universidad Autónoma de San Luis Potosí, Km 14.5 Carretera San Luis Potosí-Matehuala, Ejido Palma de la Cruz, CP 78321, Soledad de Graciano Sánchez, San Luis Potosí, Mexico; E-Mail: jcrodort@hotmail.com

**Keywords:** *Capsicum annuum* L., capsaicin, dihydrocapsaicin, pungency, temperature stress

## Abstract

The chili pepper is a very important plant used worldwide as a vegetable, as a spice, and as an external medicine. In this work, eight different varieties of *Capsicum annuum* L. have been characterized by their capsaicinoids content. The chili pepper fruits were cultivated in the Comarca Lagunera region in North of Mexico. The qualitative and quantitative determination of the major and minor capsaicinoids; alkaloids responsible for the pungency level, has been performed by a validated chromatographic procedure (HPLC-DAD) after a preliminary drying step and an opportune extraction procedure. Concentrations of total capsaicinoids varied from a not detectable value for Bell pepper to 31.84 mg g^−1^ dried weight for Chiltepín. Samples were obtained from plants grown in experimental field and in greenhouse without temperature control, in order to evaluate temperature effect. Analysis of the two principal capsaicinoids in fruits showed that the amount of dihydrocapsaicin was always higher than capsaicin. In addition, our results showed that the content of total capsaicinoids for the varieties Serrano, Puya, Ancho, Guajillo and Bell pepper were increased with high temperature, while the content of total capsaicinoids and Scoville heat units (SHU) for the varieties De árbol and Jalapeño decreased. However, the pungency values found in this study were higher for all varieties analyzed than in other studies.

## 1. Introduction

The chili is the fruit of plants that belong to the genus *Capsicum*, which is comprised of more than 200 varieties grouped into more than 30 species, out of which five are domesticated: *C. annuum* L., *C. baccatum* L., *C. chinense* Jacq., *C. frutescens* L. and *C. pubescens* Ruiz & Pav. [1]. Among them, *C. annuum* is by far the best-known and of greatest economic importance since it presents a largest distribution worldwide and it is usually consumed either raw or cooked and used as additives in the food industry [2]. The fruits are an excellent source of health-related compounds, such as ascorbic acid (vitamin C), carotenoids (provitamin A), tocopherols (vitamin E), flavonoids, and capsaicinoids [3].

**Figure 1:**
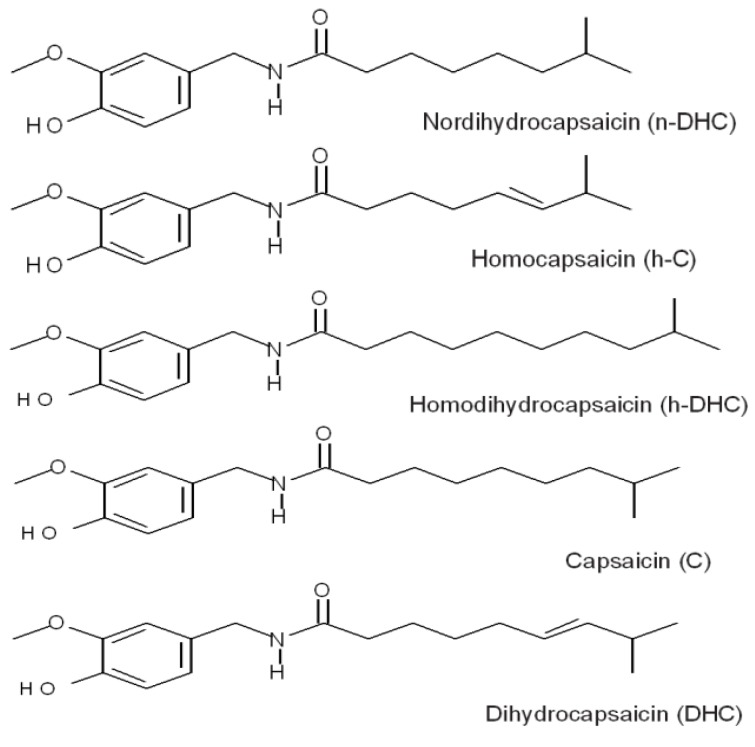
The chemical structures of the main capsaicinoids identified in this study.

The plant synthesizes and accumulates capsaicinoids, a group of alkaloids responsible for the hot or spicy flavor, which are located primarily in the tissue of the placenta adjacent to the seeds [4]. Their concentration depends on genotype, fruit maturity, and conditions of cultivation [5]. The term capsaicinoids describes a group of pungent chemical analogues which are found exclusively in chili peppers [6]. Capsaicinoids are N-vanillylamides of fatty acids. There are principally five naturally occurring capsaicinoids: capsaicin, dihydrocapsaicin, nordihydrocapsaicin, homocapsaicin and homodihydrocapsaicin (Figure 1). The most abundant and potent analogues in peppers (and consequently pepper extracts) are capsaicin and dihydrocapsaicin [7]. The only difference between them is the presence of a carbon-carbon double bond (Figure 1). Nordihydrocapsaicin, homocapsaicin and homodihydrocapsaicin are also present in pepper, but generally contribute little to the total capsaicinoid concentration and pungency of the fruits [6]. The capsaicinoids also have analgesic, anti-inflammatory, and antioxidant properties, including anticarcinogenic properties that inhibit andogen-dependent growth of breast cancer, colon, prostate and gastric adenocarcinoma [8,9,10].

Previous chromatographic methods have been reported for analytical separation, quantitation and identification of capsaicinoids by gas chromatography [11]; reverse-phase high performance liquid chromatography coupled tandem mass spectrometry (HPLC-MS) [12], capillary electrophoresis [13] and high resolution magic angle spinning-nuclear magnetic resonance (HRMAS-NMR) [14].

Mexico is the country with the greatest diversity of chili peppers. This constitutes both part of national traditions and cultural identity. In the Mexican Northwest region, fresh chili pepper types are in great demand. The harvested area is close to 143,975 hectares and average production is 16.22 t ha^−1^ [15,16]. The pepper varieties most commonly grown in Mexico are Ancho, De árbol, Guajillo, Habanero, Jalapeño, Manzano, Pasilla, Piquín and Serrano [17]. Some varieties, such as Jalapeño and Serrano, are mainly used to prepare sauces and smashed avocado (guacamole). Other chili pepper varieties, such as Piquín and De árbol, are mainly used in dry state and ground. Piquín chili pepper is also consumed pickled. Chipotle peppers (smoked Jalapeños) are very popular because of their singular taste, a little bit sweet, and can be found canned and processed.

Pungency is a key characteristic associated with members of the genus *Capsicum* and is also an important attribute of fruit quality [18]. The pungent chili varieties are grown for their food value, health-promoting properties [19]. There are five levels of pungency based on the use of Scoville heat units (SHU): non-pungent (0–700 SHU), mildly pungent (700–3,000 SHU), moderately pungent (3,000–25,000 SHU), highly pungent (25,000–70,000 SHU), and very highly pungent (>80,000 SHU) [20]. Like any subjective scale, the pungency scale is only a guide.

In this sense, the growth and pungency levels of *Capsicum* fruits are affected by a series of environmental factors [21], and the ones that have the greatest impact are: water availability, sunlight and the temperature [22]. Reduced fruit set during periods of high temperature has been documented for a wide variety of plant species including chili peppers. Therefore, high temperature constitutes one of the major hazards to agriculture because that limits the survival, productivity and geographical distribution of chili peppers in large areas of the world. Despite its importance, little research has been done on cultivation conditions that may affect its productivity and the fruit quality in semi-desert regions.

The weather of the Comarca Lagunera is semi-desertic with low atmospheric humidity and an average rainfall of 240 mm a year [23]. Temperatures tend to be more than 40 °C in the summer season, which causes both thermic and hydric stress in crops [24]. In this context, same agricultural practices are often used for different chili pepper types, although each type differ in growth, habit, and fruiting characteristics. There are few studies reporting the capsaicinoids content and proximate composition of various different cultivars of chili pepper [25,26]; however, in all these previous studies, *Capsicum* samples were purchased at local markets and most of them were found in a dry state. Thus, there is a serious lack of information about the biochemical responses of chili pepper regarding management differences. In addition, information on capsaicinoids contents of peppers commonly consumed in Northern Mexico is still scarce. The aim of this study was to investigate the effect of high temperatures on the content of capsaicinoids in different varieties of peppers in the stage named mature green, grown both in greenhouses and in the field, because in Mexico, a high percentage of chili fruits is consumed at this maturity stage [16].

## 2. Results and Discussion

**Figure 2:**
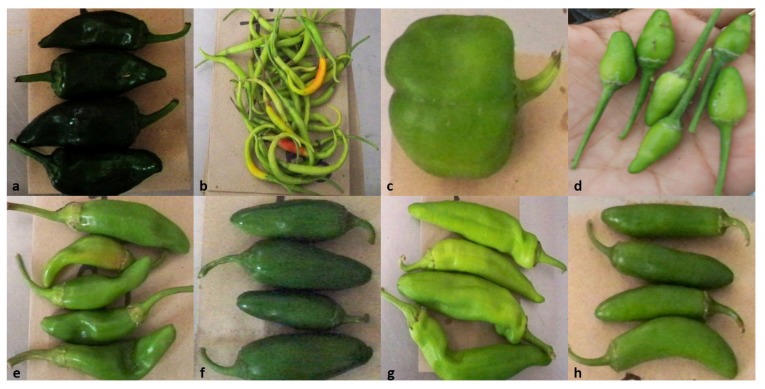
Chili samples used in this analysis. (**a**) Ancho cv. Don Matías; (**b**) De árbol; (**c**) Bell pepper cv. Cardinal; (**d**) Chiltepín; (**e**) Guajillo cv. San Luis; (**f**) Jalapeño cv. Don Julio; (**g**) Puya; (**h**) Serrano cv. Don Diego.

In order to characterize capsaicinoids in chili pepper, acetonitrile extracts were prepared from dry fruits of eight different varieties; all of them belonging to *C. annuum*. Bell pepper is a green pepper with great flavor but not pungency, included in this study as a negative reference for comparison with other chili peppers. Figure 2 illustrates the morphology of the various types of peppers used in this study.

Extracts were injected and separated by HPLC-DAD. Under the optimum conditions, calibration curves were obtained by injecting standard solutions at ten different concentrations. Each point on the calibration graph corresponded to the mean value obtained from three independent peak area measurements. Calibration curves were y = 8.824x − 2.54 for capsaicin and y = 14.312x − 2.12 for dihydrocapsaicin. The calibration curves exhibited excellent linear behavior over the concentration range of 1.5–1,200 µg mL^−1^ for capsaicin and 1.25–500 µg mL^−1^ for dihydrocapsaicin with a correlation coefficient (r) 0.9999 and 1, respectively.

The analytical method was validated according to International Conference on Harmonization (ICH) guidelines [27]. The detection limits of capsaicin and dihydrocapsaicin were 0.5 µg mL^−1^ at 222 nm and 0.75 µg mL^−1^ at 280 nm, respectively. While the quantification limits were 1.5 and 1.25 µg mL^−1^ for capsaicin and dihydrocapsaicin, respectively. The repeatability of the method was determined with a mixture of standard solution at the level of 50 µg mL^−1^ for the analytes. The intra-assay repeatability values were of 0.5 min for retention time and 5% for peak area. Whereas the inter-assay repeatability values were lower than 0.7 min for retention time and 10% for peak area. To determine the recovery of the extract of peppers, concentrations of capsaicin and dihydrocapsaicin in the range of 10–150 µg mL^−1^ were added to Bell pepper extract. Recoveries of the added capsaicin and dihydrocapsaicin were in the range 92%–102% and 94%–109%, respectively.

### 2.1. Quantification of Individual Compounds

**Figure 3:**
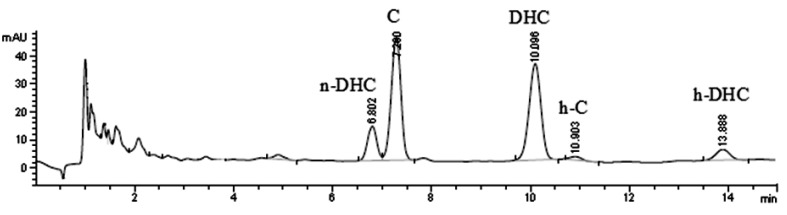
Chromatogram of Jalapeño pepper extract. Nordihydrocapsaicin (n-DHC), capsaicin (C), dihydrocapsaicin (DHC), homocapsaicin (h-C) and homodihydrocapsaicin (h-DHC).

Capsaicinoids analysis by high performance liquid chromatography (HPLC) currently allows a precise determination of the nature and quantity of these alkaloid compounds [28,29]. Fruit of different varieties showing a diversity of capsaicinoids that includes capsaicin, dihydrocapsaicin, homocapsaicin, homodihydrocapsaicin and nordihydrocapsaicin, were analyzed by HPLC-DAD to compare the total level and composition of their capsaicinoids at the green stage. Figure 3 shows the chromatogram where it was possible to identify the five main capsaicinoids that are found in the Jalapeño pepper. Quantifications were based on average peak areas of injections of 20 µL obtained from external standard solutions of capsaicinoids prepared in acetonitrile. Capsaicin and dihydrocapsaicin were identified and quantified using standard compounds. Under these conditions, retention times (Rt) were 7.0 and 10.0 min for capsaicin and dihydrocapsaicin respectively.

Nordihydrocapsaicin, homodihydrocapsaicin and homocapsaicin were tentatively identified by their consistent retention time and coelution on the basis of its chromatographic behavior on a C_18_ column as reported by other authors [30,31]. Nordihydrocapsaicin and homodihydrocapsaicin were quantified using dihydrocapsaicin, because commercial standards of these particular compounds are not available. Values were reported as equivalents of dihydrocapsaicin. Homocapsaicin was quantified using capsaicin and values were reported as capsaicin equivalents by the same cause than the compounds previously mentioned.

With the samples obtained from field conditions, we found that in all pepper fruits, the capsaicin is accumulated at various levels with the exception of the Bell pepper. Concentrations of capsaicin found were in the range of 0.17–15.36 mg g^−1^ of dried fruit, the capsaicin contents decreased as follow: Chiltepín, Jalapeño, De árbol, Serrano, Puya, Ancho and Guajillo (Table 1). However, dihydrocapsaicin was found to be the major component (41%–57%) of the total capsaicinoids in the green fruits of almost all varieties. Concentrations observed were in the range of 0.60–13.39 mg g^−1^ of dried fruit for dihydrocapsaicin. The levels of other capsaicinoids including nordihydrocapsaicin, homodihydrocapsaicin and homocapsaicin varied from 1% to 38%, and the homocapsaicin never was detected above 2% of the total capsaicinoids in most of varieties studied. Chiltepín had twice more capsaicin and dihydrocapsaicin than De árbol, and about 1.5 times than Jalapeño. In terms of the content of main capsaicinoids, peppers De árbol and Chiltepín are exceptional among varieties as they displayed levels of capsaicin ranging from 82%–90%. Similarly high levels of homodihydrocapsaicin were detected in samples of Ancho and Guajillo with levels of 31% and 38%, respectively. Whereas in Bell pepper capsaicinoids are undetectable in fruits of plant grown in open field.

The ratio of capsaicin to dihydrocapsaicin is generally 1:1 and 2:1 [32]. Manirakiza *et al*. [33] indicated that in *C. annuum* the ratio is close to 1:1, while in *C. frutescens* the ratio is 2:1. Although capsaicin is usually the most abundant capsaicinoid, the contents of dihydrocapsaicin were higher than those of capsaicin in about all varieties (Table 1). Therefore the C:DHC ratio to these varieties were 1:2. Similar patterns, with dihydrocapsaicin being the predominant capsaicinoid, have been previously reported in other varieties of *C. annuum* and *C. pubescens* [34,35]. As well, Cruz-Pérez *et al*. [36] observed a greater concentration of DHC in samples of *C. pubescens*.

The level of total capsaicinoids was significantly higher in all peppers. Highly significant differences were found in the capsaicinoids concentrations. The lowest value was observed in Guajillo, and highest in Chiltepín. As shown in Table 1, the Chiltepín variety accumulated the highest level of total capsaicinoids (31.84 mg g^−1^ dry weight), both De árbol and Jalapeño varieties accumulated total capsaicinoids at lower levels compared with Chiltepín, but the two varieties showed higher levels than varieties Puya and Serrano. Both Guajillo and Ancho accumulated total capsaicinoids at lower levels than Puya and Serrano. None capsaicinoid were detectable in Bell pepper in this condition. 

These values are out in the range of those reported by different authors for peppers Chiltepín [11], Jalapeño and Serrano [19]. For example, it has been reported that the fruit of Chiltepín in its immature form has very low levels of capsaicin (0.64 mg g^−1^) and dihydrocapsaicin (0.37 mg g^−1^). Those values correspond to 24 and 36 times lower than obtained in this study. In addition, in the case of other peppers, a similar trend was observed in De árbol pepper, it has been reported that levels of capsaicin and dihydrocapsaicin were 1,293.36 mg kg^−1^ and 641.74 mg kg^−1^, respectively [15]. In contrast, our study shown that variety De árbol reached about among in each 4 and 10 times more among in each capsaicinoid, 5,218 mg kg^−1^ for capsaicin and 6,247 mg kg^−1^ for dihydrocapsaicin. In the present study, the results obtained indicate an increase in the capsaicin content of 22 times and in dihydrocapsaicin the increase was of 34 times. These results are in agreement with Garcés-Claver *et al*. [34] who found that some *C. annuum* cultivars contain higher concentration of dihydrocapsaicin than the amounts found in *C. chinense* (Habanero). This variation is explained by the effect of regional varieties and environment conditions beside the genetic factors.

Despite the fact that the material used in this study were dry fruits and in green mature stage (Figure 1) the SHU ranged between 12,546 for Guajillo and 462,884 for Chiltepín (Table 1), thus the majority of peppers analyzed have a moderate and high hotness, however this levels could be higher in fruits in red mature stage. On the basis of the hotness scale, capsaicinoids content in native pepper morphotypes of *C. chinense* and *C. annuum* from Yucatan, Mexico, range from 1,000 SHU to greater than 235,000 SHUs [37,38,39]. Other peppers extremely pungent include Naga chili, it was recorded at 1,001,304 SHUs, whereas Red Savina Habanero recorded 248,556 SHUs [40]. In this study, the results showed that Chiltepín surpassed the pungency levels reported for Habanero pepper, and although Jalapeño (280,572 SHU) and De árbol (184,610 SHU) registered a pungency lower that orange Habanero (357,729 SHU) [41], these values surpassed the pungency levels reported for other authors [15,19,42].

### 2.2. Effect of High Temperature on Capsaicinoids Content

High temperature stress is defined as the rise in temperature beyond a critical threshold for a period of time sufficient to cause irreversible damage to growth and development of a plant [43]. The growth and development of plants involves a countless number of biochemical reactions, all of which are sensitive to some degree to temperature [44]. Consequently, the plant responses to high temperature vary with the extent of the temperature increase, its duration, and the plant type. Worldwide, extensive agricultural losses are attributed to heat, often in combination with drought or other types of stress [45].

Pepper plants are originated from tropic regions and require high temperature conditions for their development. Consequently, the optimum growth temperature is between 25 and 30 °C. Temperature changes affect a variety of physiological functions and morphological development [46]. Very little fruits occurs when temperatures are above 30 °C during the day or below 15 °C at night and usually are small and poorly shaped. Some of the small fruited pungent are more tolerant to high temperature fruit set problems than type Bell pepper. Pungent peppers, such as Jalapeño, grow well in hot weather and can often produce fruit throughout the summer. The contents of capsaicinoids are thought to vary under water or nutritional stress conditions. Thus, high temperatures could have important effects on capsaicin content at different fruit maturity stages in Bell pepper cultivars [47].

Analysis of capsaicin and dihydrocapsaicin of seven varieties of *C. annuum* under greenhouse conditions indicated that the concentrations and relative proportions of capsaicinoids varied significantly between cultivars with high temperature. Serrano peppers contained the greatest concentrations of total capsaicinoids (Table 2) compared to other cultivars tested. Concentrations of five mains capsaicinoids are presented in Table 2. Concentrations of capsaicin varied among cultivars from 0.38 mg g^−1^ dry weight in Ancho sample to 4.76 mg g^−1^ dry weight in Serrano (Table 2). Concentration of dihydrocapsaicin varied among varieties from 0.63 mg g^−1^ in Ancho pepper to 10.14 mg g^−1^ in Serrano. Concentrations of total capsaicinoids varied among cultivars from 0.41 mg g^−1^ (only homodihydrocapsaicin) in Bell pepper to 18.048 mg g^−1^ in Serrano. Therefore, Serrano pepper contained the greatest concentrations of each of the pungent capsaicinoids (capsaicin, dihydrocapsaicin and nordihydrocapsaicin) among all the cultivars tested under high temperature.

**Table 1:** Content of capsaicinoids in chili samples grown in an environment without thermal stress. Nordihydrocapsaicin (n-DHC), capsaicin (C), dihydrocapsaicin (DHC), homocapsaicin (h-C) and homodihydrocapsaicin (h-DHC); SHU = Scoville heat units. Content of nordihydrocapsaicin and homodihydrocapsacin were calculated as equivalents of dihydrocapsaicin; the content of homocapsaicin was calculated as equivalents of capsaicin. Means in the same column followed by diﬀerent letters (a–e) were signiﬁcantly diﬀerent by Tukey’s test at *p* ≤ 0.05. ND = not detected.

Sample	n-DHC		C		DHC		C:DHC ratio	h-C		h-DHC		Total capsaicinoids	SHU
	mg g^−1^	%	mg g^−1^	%	mg g^−1^	%		mg g^−^^1^	%	mg g^−1^	%	mg g^−1^	
	dry weight		dry weight		dry weight			dry weight		dry weight		dry weight	
Ancho cv. Don Matias	0.25 ± 0.01 ^ab^	13	0.29 ± 0.03 ^a^	15	0.77 ± 0.04 ^a^	41	1:2.7	ND	−	0.58 ± 0.12	31	1.88 ± 0.05 ^a^	19,335.7 ± 1,133.7 ^a^
De árbol	1.07 ± 0.01 ^c^	8	5.22 ± 0.16 ^b^	37	6.25 ± 0.30 ^c^	45	1:1.2	0.13 ± 0.11	1	1.29 ± 0.77	9	13.96 ± 0.29 ^c^	194,591.9 ± 6,852.6 ^c^
Bell pepper cv. Cardinal	ND	−	ND	−	ND	−	ND	ND	−	ND	−	ND	ND
Chiltepín	2.17 ± 0.21 ^d^	7	15.36 ± 1.67 ^d^	48	13.39 ± 1.60 ^e^	42	1:0.9	0.22 ± 0.07	1	0.69 ± 0.19	2	31.84 ± 0.75 ^e^	483,089.3 ± 54,336.8 ^e^
Guajillo cv. San Luis	0.12 ± 0.03 ^a^	8	0.17 ± 0.01 ^a^	12	0.61 ± 0.11 ^a^	42	1:3.6	ND	−	0.56 ± 0.16	38	1.46 ± 0.08 ^a^	13,704.8 ± 2,275.7 ^a^
Jalapeño cv. Don Julio	2.48 ± 0.28 ^d^	12	8.03 ± 0.37 ^c^	38	9.39 ± 0.41 ^d^	45	1:1.2	0.16 ± 0.13	1	0.97 ± 0.14	5	21.03 ± 0.27 ^d^	303,602.5 ± 8,722.0 ^d^
Puya	0.55 ± 0.06 ^b^	11	1.18 ± 0.07 ^a^	25	2.32 ± 0.22 ^ab^	48	1:2.0	0.08 ± 0.01	2	0.68 ± 0.04	14	4.80 ± 0.08 ^ab^	61,526.0 ± 5,117.4 ^ab^
Serrano cv. Don Diego	0.53 ± 0.04 ^b^	8	1.52 ± 0.01 ^a^	24	3.54 ± 0.01 ^b^	57	1:2.3	ND	−	0.64 ± 0.09	10	6.25 ± 0.04 ^b^	86,427.2 ± 635.4 ^b^

**Table 2:** Content of capsaicinoids in chili samples grown in an environment with thermal stress. Nordihydrocapsaicin (n-DHC), capsaicin (C), dihydrocapsaicin (DHC), homocapsaicin (h-C) and homodihydrocapsaicin (h-DHC); SHU = Scoville heat units. Content of nordihydrocapsaicin and homodihydrocapsacin were calculated as equivalents of dihydrocapsaicin; the content of homocapsaicin was calculated as equivalents of capsaicin. Means in the same column followed by diﬀerent letters (a–e) were signiﬁcantly diﬀerent by Tukey’s test at *p* ≤ 0.05. ND = not detected.

Sample	n-DHC		C		DHC		C:DHC ratio	h-C		h-DHC		Total capsaicinoids	SHU
	mg g^−1^	%	mg g^−1^	%	mg g^−1^	%		mg g^−1^	%	mg g^−1^	%	mg g^−1^	
	dry weight		dry weight		dry weight			dry weight		dry weight		dry weight	
Ancho cv. Don Matias	0.44 ± 0.26 ^ab^	22	0.38 ± 0.05 ^a^	18	0.63 ± 0.00 ^a^	31	1:1.7	ND	−	0.60 ± 0.04	29	2.05 ± 0.09 ^a^	20,275.2 ± 2815.5 ^d^
De árbol	0.64 ± 0.05 ^ab^	7	4.20 ± 0.07 ^d^	44	4.19 ± 0.04 ^c^	45	1:1.0	0.04 ± 0.02	0.5	0.33 ± 0.06	4	9.40 ± 0.50 ^c^	141,072.7 ± 2,091.9 ^d^
Bell pepper cv. Cardinal	ND	−	ND	−	ND	−	ND	ND	−	0.41 ± 0.08	100	0.41 ± 0.08 ^a^	ND
Guajillo cv. San Luis	0.27 ± 0.02 ^a^	6	1.32 ± 0.16 ^b^	28	2.83 ± 0.27 ^b^	60	1:2.2	ND	−	0.33 ± 0.08	7	4.74 ± 0.13 ^b^	69,210.5 ± 7,228.8 ^b^
Jalapeño cv. Don Julio	1.19 ± 0.08 ^c^	15	2.35 ± 0.11 ^c^	29	4.18 ± 0.27 ^c^	51	1:1.8	ND	−	0.40 ± 0.13	5	8.11 ± 0.15 ^c^	116,158.5 ± 6,534.7 ^c^
Puya	0.90 ± 0.04 ^bc^	15	1.54 ± 0.26 ^b^	27	3.37 ± 0.82 ^bc^	58	1:2.2	ND	−	ND	−	5.81 ± 0.37 ^b^	87,440.1 ± 19,221.8 ^bc^
Serrano cv. Don Diego	2.47 ± 0.08 ^d^	14	4.76 ± 0.22 ^e^	26	10.14 ± 0.56 ^d^	56	1:2.1	0.36 ± 0.03	2	0.31 ± 0.14	2	18.05 ± 0.21 ^d^	262,916.3 ± 13,306.4 ^e^

High temperatures can be a negative factor in the accumulation of capsaicinoids in certain varieties of chili peppers, this study showed that the varieties most affected were Jalapeño and De árbol peppers as they lose 61.5% and 32.5% of total capsaicinoids, respectively. Moreover, the temperature rise in greenhouse favored the accumulation of total capsaicinoids in varieties Guajillo and Serrano and corresponding to an increase 3-fold. Furthermore, the Puya variety showed a slight increase of 21% in the amount of total capsaicinoids; while having a slightly positive effect on Ancho with an increase of 8.6%. These data demonstrate that the responses of the peppers did not show a homogeneous behavior. In literature it has been reported that levels of capsaicinoids increase in spicy peppers varieties more than in sweet peppers when the temperature rises in the place where cultivated, however, our results show a decrease in the amount of capsaicinoids in Jalapeño and De árbol varieties.

Based on results obtained in this study, it is tempting to postulate that capsaicinoids accumulation could be compensatory mechanism between different metabolic pathways in response to thermal stress. Tiwari *et al*. [48] observed the decrease in the amount of capsaicin and dihydrocapsaicin of Naga chili by about 50% when cultivated at Gwalior, India indicating that lower levels of humidity and rainfall were not favorable for this crop. Estrada *et al*. [49] demonstrated that the environmental conditions, such as water stress, have a strong effect upon the accumulation of capsaicinoids in Padrón peppers fruits, which is the result of competition between biosynthesis of capsaicinoids and phenylpropanoid metabolites.

Despite our understanding of capsaicinoids biosynthesis, we know very little about the capsaicinoids-accumulation behavior of peppers under different environmental conditions. Phenolic intermediates can influence the biosynthesis of capsaicinoids [50]; for instance, it has been demonstrated that 8-methylnonenoic acid could have an important regulatory role in the capsaicinoids biosynthesis pathway [51]. The synthesis of flavonoids may converge with the capsaicinoids pathway during pepper maturation [52]. According to a study of Sukrasno and Yeoman [53], capsaicinoid accumulation is parallel to the disappearance of flavonoids together with an accumulation of lignin-like substances. Lee *et al*. [54], studying 12 pepper cultivars, found that the hotness (pungency) index, which represented total capsaicinoid content, was not inversely associated with total flavonoid content. Moreover, mild peppers did not have greater flavonoid concentration than hot ones. They said that biosynthesis of flavonoids may be completed with capsaicinoids synthesis in phenylpropanoid metabolism, and each pepper type may regulate flavonoid synthesis differently.

The capsaicin biosynthesis involved the activity of capsaicin synthase in the placental tissue and capsaicin synthesis might be increased in high temperature, nutrient solution and advancement of fruit maturity. Capsaicinoid biosynthesis and accumulation is a genetically determined trait in chili pepper fruits as differences in pungency. Furthermore, this characteristic is also developmentally and environmentally regulated. Pungency results from the accumulation of the capsaicinoid alkaloids in the placenta of the fruit, and is unique to the *Capsicum* genus. The presence or absence of pungency is controlled by one locus, Pun1 (formerly C). The candidate gene under Pun1 was identified from genes that were differentially expressed in pungent versus non-pungent fruits. This candidate gene, AT3, encodes a protein with high homology to an acyltransferase and is tightly linked to Pun1 [55] the mechanism by which AT3 is capsaicin synthase, the last enzyme in the capsaicinoid biosynthesis pathway, postulated to be an acyltransferase.

A study has shown that capsaicin and dihydrocapsaicin diminish after cellular disruption of the fruits, which is apparently due to the dependence of oxidation to the temperature [56]. There is some evidence that peroxidase isoenzymes may directly be involved in the capsaicinoid metabolism, since the vanillyl moiety of capsaicin is readily oxidized by these enzymes [57]. Thus, the increase in capsaicinoids content always coincided with a low or decreased peroxidase activity, and the decrease in their concentrations always coincided with a high or increased enzyme activity [58].

Another factor significantly influencing the total capsaicinoids content is climate. Chili peppers may become more or less pungent if they are stressed. Two types of stress that affect the pungency of peppers are having a high average temperature outside, and insufficient watering. Another form of stress is overwatering. It does not matter what type of stress will increase or decrease the pungency of the fruit. According to Saha *et al*. [59] four Bell pepper varieties differed significantly the carotenoids content and these genotypes were considered to be heat sensitive. Habanero pepper plants under water stress had a lower height, root dry weight, and root/shoot relation than control plants, which were irrigated daily. However, fruit growth and production were unaffected by water stress. Capsaicin and dihydrocapsaicin concentrations increased in fruits of stressed plants compared with control plants, and this effect was correlated with fruit age. However, capsaicin synthase activity was reduced in response to water stress, and this effect depended on both stress severity and fruit age [60].

In this context, it is not clear how high temperature affect the synthesis and accumulation of capsaicinoids, it is necessary to carry out further studies with use HRMAS-NRM method for determination of metabolites of pepper, which is useful for metabolomics analysis [14].

## 3. Experimental

### 3.1. Chemicals

All the reagents and solvents used were of analytical or HPLC grade and were purchased from Sigma-Aldrich (St. Louis, MO, USA).

### 3.2. Samples and Cultivar Conditions

Eight varieties of pepper (*Capsicum annuum*) were evaluated, seven of whom were from Culiacan, Sinaloa, Mexico: Ancho cv. Don Matías, De árbol, Bell pepper cv. Cardinal, Guajillo cv. San Luis, Jalapeño cv. Don Julio, Puya, and Serrano cv. Don Diego; and Chiltepín at green stage of maturity.

For germination, the seeds of the different genotypes were placed on Styrofoam trays of 200 holes disinfected with chlorine 0.05% on 16 January 2012. Peat moss and vermiculite in a ratio of 2:1 (v/v) were used as substrate. A seed was placed per each well at 0.5 cm deep. The trays were covered with plastic wrap for 72 hours to ensure uniform germination; transplant took place on March 18, 2012.

The transplant was performed in a greenhouse and experimental field of the Facultad de Agricultura y Zootecnia of the Universidad Juarez del Estado de Durango (FAZ-UJED) located on 25°47'1'' N and 103°20'58'' W, in the north-center of Mexico in the region named La Comarca Lagunera.

In both sites, greenhouse and experimental field, we used 4.3 ton ha^−1^ of vermicompost as substrate. The density of plants was 8 plants m^−2^. Each plant in the greenhouse was placed in a polystyrene bag with a capacity of 10 kg, each bag was filled with vermicompost and sterile sand, in experimental field each plant was placed directly on the soil. In both sites, plants were watered drip to field capacity. The minimum temperature in the experimental field was 33 °C and the maximum was 40 °C. In the greenhouse, temperature and humidity were not controlled, so that the minimum and maximum temperatures were 40 and 48 °C respectively.

Samples collected were dried in an oven at 65 °C for 72 h. The dry weights of the fruit were 5.32 g and 2.6 g in the samples of experimental field and greenhouse, respectively. Moisture loss in samples from both sites was 85%.

### 3.3. Capsaicinoids Extraction

The capsaicinoids were extracted from the dried fruits following the methods proposed by Al Othman *et al*. [61] and Parrish [62] slightly modified as follows: briefly, the dried fruits were powdered and 1 g were treated with 10 mL of acetonitrile at the temperature of 65 °C along 20 min under sonication in an ultrasonic bath with a working frequency of 35 kHz. The extracts were evaporated to dryness at 60 °C, resuspended in 0.5 mL of acetonitrile, and filtrated through 0.45 µm cellulose acetate membrane filter (GVS Filter Technology, Indianapolis, IN, USA). Samples were stored at −20 °C until they were analyzed.

### 3.4. Capsaicinoids analysis by HPLC-DAD

The analyses of capsaicinoids were performed by HPLC-DAD (Agilent 1200, Agilent Technologies Palo Alto, CA, USA) employing a reversed phase column Kromasil Eternity-5-C18 (4.6 × 150 mm) with Pre-column (SUPELCO Analytical, Sigma-Aldrich) at 25 °C. Elution was performed with an isocratic mixture of water:acetonitrile 50:50. Detection was set at 222 and 280 nm. Injection volume was 20 µL. All peaks related to capsaicinoids were eluted in about 15 min. Quantitative analysis was performed following the external standard method. Calibration curves were built by injecting, in triplicate, ten increasing concentrations of standard. Nordihydrocapsaicin and homocapsaicin were quantified using the calibration curve of dihydrocapsaicin, since no performed were commercially available. All measures were performed in triplicate.

### 3.5. Pungency

Capsaicinoid contents were converted to Scoville heat value (SHV) by multiplying the pepper dry weight capsaicinoid concentration in parts per million (ppm) by the coefficient of the heat value for each compound, 9.3 for nordihydrocapsaicin and 16.1 for both capsaicin and dihydrocapsaicin [63]:

Total SHU = [C(ppm) + DHC(ppm)] × 16.1] + [n-DHC(ppm) × 9.3]

### 3.6. Statistical Analysis

Experimental data are shown as the mean ± standard error of assay run in triplicate for capsaicinoids content. The statistical test was an ANOVA, for which we used SPSS version 15.0 for Windows.

## 4. Conclusions

This study evidenced a great variability in capsaicinoids and showed that the increase in temperature of growing conditions had a significant effect on the type and total capsaicinoids in fruits of sweet and hot pepper grown in Comarca Lagunera. Nordihydrocapsaicin, homocapsaicin and homodihydrocapsaicin were always present at low concentrations when compared to capsaicin and dihydrocapsaicin. In all cases, dihydrocapsaicin concentration was greater than that of capsaicin. Future studies into the activity and expression of some peroxidases and capsaicin synthase are needed to evaluate their effects on capsaicinoids accumulation under high temperature condition in Guajillo, Serrano, Jalapeño and De árbol cultivars.
